# Social Networking Sites and Youth Transition: The Use of Facebook and Personal Well-Being of Social Work Young Graduates

**DOI:** 10.3389/fpsyg.2020.00230

**Published:** 2020-02-18

**Authors:** Joaquin Castillo de Mesa, Luis Gómez-Jacinto, Antonio López Peláez, Amaya Erro-Garcés

**Affiliations:** ^1^University of Málaga, Málaga, Spain; ^2^National University of Distance Education, Madrid, Spain; ^3^Universidad Pública de Navarra, Pamplona, Spain

**Keywords:** digital skills, well-being, life satisfaction, self-esteem, social capital

## Abstract

Research on youth transitions, and the well-being of young people, has to take into consideration the digital context in which they are immersed. Digital interaction of young people increase year by year, social networking sites play a key role in their personal and professional relationships, and a very high percentage of jobs require digital skills. According to Eurostat (2019), participating on social networking sites (one of the most common online activities in the EU-28), is growing every year [more than half (56%) of individuals aged 16–74 used the internet for social networking sites], and this percentage increases among the younger generations. In this article, we present the results of our research on the digital skills and well-being of young people on Facebook, based on a survey with a sample of 126 young people graduated from the University of Málaga (School of Social Work) (Spain). Based on certain scales, the level of digital skills that students have on Facebook was measured, considering strategic aspects for information search, level of use and presence of Facebook in life, maintenance of relations and tolerance to diversity. Variables of psychosocial well-being were also measured (social capital, self-esteem, life satisfaction, and personal well-being). Variables of digital skills on Facebook were subsequently related to well-being variables. Results show that certain digital skills relate to the well-being of young people. In this sense, we deem it crucial to develop education policies that could provide young graduates with general digital skills to be used on social networking sites.

## Introduction

We are immersed in a digital society, in which our social relationships, communication, education, leisure and work are transformed. Our vital trajectories, our autonomy, and our well-being, are influenced by digitalization. To analyze the different dimensions of well-being such as environmental mastery or positive relationships (Ryff, 2019), it is necessary to take into account the technological environment and how young people participate, learn and interact on social networking sites. The new generations, intensive users of technology, are considered digital natives, and therefore digital competences play a key role in their well-being. Consequently, institutions and companies are focused on the development of these skills (Picatoste et al., 2018). To quote an example, the Council of the European Union included digital skills in their conclusions related to education and training whereas the Europe 2020 strategy considers Information and Communication Technologies (ICTs) as a key element in the education reform.

Therefore, technology can be considered a driver for well-being as technology impacts on the experience of young across the world (Collin and Burns, 2009). A plethora of studies highlight the role of digital skills as drivers for well-being in Youth. ICTs promote well-being facilitating informal learning, building digital identities, improving competences required at the workplace or promoting meta-social skills, among others (e.g., Sánchez-Navarro and Aranda, 2013; Goldhammer et al., 2016; Martinovic et al., 2019). Additionally, identity is built during the transition to adulthood and digital skills can perform a crucial role in this process. In this sense, Mannerström (2019) showed that identity formation was related to digital practices and competencies. Along with this line, scholars suggest that there is a positive relationship between youth empowerment and certain uses of digital tools (Middaugh et al., 2017). Furthermore, ICTs enable well-being in the case of physical or intellectual disabilities (Pacheco et al., 2019) and are particularly important to support at-risk youth (Helsper and Van Deursen, 2015; Pienimäki, 2019). These benefits justify the inclusion of digital skills in formal and informal education. Nevertheless, a view from the dark side should be also evidenced. Equity and inclusion problems (Pagani et al., 2016), cyber-aggression (Mishna et al., 2018), technology addiction (Lachmann et al., 2018; Wang et al., 2018) or the negative effects of ICTs on learning and academic results (Hawi and Samaha, 2016) have been underlined as disadvantages of technology related to Youth.

From our point of view, social networking sites play a key role in the lives of young people, and digital skills are key to strengthening their vital trajectories, and therefore relevantly affect the different dimensions of well-being (Ryff, 2018). In this digital environment, below we present the results of our research on digital skills and the well-being of young people on Facebook, with a sample of 126 young graduates from the University of Málaga (School of Social Work) (Spain). Based on certain scales, the level of digital skills that students have on Facebook was measured, considering strategic aspects for information search, level of use and presence of Facebook in life, maintenance of relations and tolerance to diversity. Variables of psychosocial well-being (SW) were also measured [social capital, self-esteem, life satisfaction and personal well-being (PeW)]. Variables of digital skills on Facebook (DSF) were subsequently related to well-being variables. Results show that certain digital skills relate to the well-being of young people. In this sense, we deem it crucial to develop education policies that could provide young graduates with general digital skills to be used in social networking sites.

### Social Work Young Graduates and Social Networking Sites

Connectedness between people and organizations has progressively and exponentially increased thanks to social networking sites, which have enabled interactive dynamics that were unimaginable until recently. Social networking sites have become a global social phenomenon. Facebook is one of the main personal-profile networking sites and in 2016 it announced having reached the symbolic figure of two billion active users on a monthly basis. This makes Facebook the most used social networking site, as it gathers more than one fourth of the population worldwide. In Europe, Facebook enables five billion social connections (Filiz et al., 2016). More than 75% of the population use personal-profile networking sites on a daily basis and the average time spent has increased until reaching an average of almost 2 h per day (Roth, 2018). In sum, the number of users worldwide, the frequency of connection and the time spent make of Facebook a parallel universe for socialization (Wilson et al., 2012).

The presence of social networking sites is remarkably high in all sectors of society, and it is even higher when it comes to young people (Duggan and Smith, 2013). Young adults between 18 and 35 years reported being active on these sites over the past years (Pew Research Center, 2014). Young graduates tend to use social networking sites more intensively (Steinfield et al., 2008). They have grown up with these social technologies and they are now being called “digital natives” (Prensky, 2001). However, young people do not constitute a monolithic group with universal talents to use these digital means. On the contrary, their relation with digital technologies is very varied (Selwyn, 2009). Because it is an emergent phenomenon, young people have adopted and use social networking sites spontaneously. Using these sites allows them to keep their relationships with friends (Wang and Edwards, 2016) and create new ones (Levine and Stekel, 2016), amongst other purposes. Nevertheless, these sites can also have some harmful effects or lead to deviant behaviors, particularly when there is a lack of training on the effects certain types of uses can imply.

Academic literature has reached a certain consensus on the fact that the impact online communication can have on well-being depends on each user’s aim, the nature of the communication exchange and the closeness between nodes (Burke and Kraut, 2013). This approach, which focuses on the importance of the type of use, that is, “for what purpose,” suggests that the different ways in which the population uses these means depend on the digital skills individuals have (Van Deursen and Van Dijk, 2015).

Social Work graduates and Social Work as a discipline cannot remain external to the impact of this phenomenon, particularly when it comes to the increase of socialization on social networking sites. This is mainly due to the fact that these sites promote one of the main activities of Social Work, which is to build relations. Addams (1910), foremother of Social Work and visionary, gave great importance to the construction and improvement of social relations between individuals as a way to face adversity. This is the reason why various Social Work institutions such as the National Association Social Work et al. (2017) are encouraging social workers to acquire the necessary digital skills in order to be able to use digital means to find solutions to social problems and empower citizens.

### Digital Skills on Facebook

Having or not digital skills determines users’ access to resources, thus empowering those who have the appropriate skills to benefit from the potentialities of digital means and leaving behind those who do not know how to leverage such advantages (Van Dijk, 2006). Digital skills are considered as “the capacity to respond pragmatically and intuitively to challenges and opportunities in a manner that exploits de Internet’s potential” (DiMaggio et al., 2004, p. 378). It is also defined as the “user’s capacity to find content on the Internet in an effective and efficient manner” (Hargittai, 2005, p. 372).

Digital skills can be analyzed and conceptualized according to various levels. One of these levels looks at operational abilities (Steyaert, 2002). These abilities refer to knowledge, interaction and use of applications and devices. Van Dijk (2005) defines these abilities as those used to operate computers – currently also smartphones – and which relate to hardware and software networks. These digital skills refer to the ability to handle the profuse amount of resources at hand, which is also known as hypermedia (Lee et al., 2005). Digital skills are key to search, select, process and apply means to an environment which is overloaded with opinions (Van Dijk, 1999). Hargittai and Hsieh (2010) measured these skills through a scale that considers knowledge of the language and use of basic functions of the Internet (PDF, JPG, Favorites, Reload, etc.). In the online universe of Facebook, these functions are constituted by Facebook’s language and functions (Timeline, Pages, Groups, Lists, etc.).

At a secondary level, digital skills concerning information search are considered. These skills refer to actions taken by users to satisfy their information needs (Jenkins, 2006). Knowing how to look for information by using applications and services on the Internet implies a certain level of skills to filter information (Marchionini and White, 2007) and awareness about the fact that the digital fingerprint left by the use of browsers and applications leads to be suggested specific personal profiles, products or recommended advertisements.

Hargittai and Hsieh (2010) established a scale comprising two types of activities on Facebook, making a distinction between actions related to strong ties (seeing friends’ pictures, sharing photos, sending private messages, making plans, etc.) and actions related to weak ties (seeing pictures from unknown people, meeting new friends, sharing information on a group, etc.). In order to perform these actions to achieve a specific goal, strategic digital skills are required (Correa, 2016). Optimal socialization on digital means can be key for users to feel part of the same community, thus promoting various forms of mutual support and carry out projects and new initiatives (Ellison et al., 2007).

Given the fact that social reality mirrors the offline reality (Subrahmanyam et al., 2008; Dunbar et al., 2015; Gillani et al., 2018), specific abilities allowing the development of appropriate connectedness patterns are required. These patterns must include certain tolerance to diversity, which would imply being surrounded by other people who might not share our own perspective and opinions about the world, thus avoiding being immersed in “filter bubbles” or “echo chambers” (Pariser, 2011). Hence, redundancy of content and relations leading to tribal mentality and degradation of online content’s quality, security and diversity would be avoided (Gillani et al., 2018).

Finally, Jenkins-Guarnieri et al. (2013) established a scale to measure the presence of Facebook in people’s lives. This scale comprises variables that measure how people feel when they are not using Facebook or what is the role played by Facebook in people’s lives, amongst others.

### Use of Facebook and Social Capital

Feeling connected to other people is considered an “essential human motivation” (Baumeister and Leary, 1995, p. 497). People use social networking sites massively and highly frequently because they feel a need to connect and to be in contact with others (Ellison et al., 2007; Quan-Haase et al., 2017). These sites are mainly used to maintain or strengthen offline relations, rather than meeting new people (Ellison et al., 2007; Quan-Haase and Young, 2010), and they faithfully mirror socialization in the offline reality (Dunbar et al., 2015). Socialization on social networking sites and the development of communities of support and learning (Hurt et al., 2012) promote the creation of information and knowledge that is spread through the Internet (Siemens and Weller, 2011; Dron and Anderson, 2014). Using them can help satisfy the needs for social relations and increase social capital (Gosling, 2009). In particular, there is proof that Facebook helps young people improve social capital (Grieve et al., 2013).

Bourdieu (1986) defined social capital as the set of resources (current or potential) that are embedded in our social networks and which can be accessed or mobilized when needed. This concept can be analyzed from different approaches. There are different forms of social capital. At an individual level, social capital is often divided between “bridging” and “bonding” (Putnam, 2000; Williams, 2006). Bonding social capital (BOSC) is found in individuals who have very close relationships and are emotionally close and it provides emotional support or access to scarce resources (Steinfield et al., 2008). On the contrary, “bridging” social capital is found in individuals who have sporadic contact and it provides support for information and more diverse advice (Ellison et al., 2007). Bridging social capital (BSC) implies reaching more diverse information, being exposed to new ideas and have greater willingness to try different things. This form of social capital is related to well-being rates, such as self-esteem and life satisfaction (Bargh and McKenna, 2004; Huppert et al., 2004).

### Use of Facebook and Self-Esteem

Particularly during transitions between the different stages of life and as a reaction to situations and events, young people have a vital need to maintain and/or reach self-esteem. Rosenberg (1965) defined self-esteem as negative and positive attitudes toward oneself. Self-esteem comprises all inner beliefs about ourselves. Kraut et al. (2002) formulated some hypotheses relating self-esteem, social capital and satisfaction with life. One of them, the so-called “social compensation,” explains that people with low self-esteem compensate for their difficulties by socializing on the Internet.

The second hypothesis, known as “the rich become richer” assumes that people with high levels of self-esteem also feel highly satisfied when they use the Internet; they are active online and have large amounts of friends. This means that those individuals who handle themselves well in the offline world will do so in the online world. Zywica and Danowski (2008) proved both hypotheses with a group of American students in the context of Facebook. They identified two groups of users: the first group comprised extroverted students, with high self-esteem and who were popular both in the offline and online worlds; the second group comprised introverted students, with low self-esteem and who tried to compensate for their lack of popularity in the offline world by being very active on Facebook. This might explain why Facebook users with low and high self-esteem use social networking sites.

### Use of Facebook and Life Satisfaction

In the last decade, various studies have explored how the use of Internet could be related to psychological and social well-being, leading to diverse results (Kraut et al., 1998, 2002; McKenna and Bargh, 2000; Nie, 2001; Shaw and Gant, 2002; Valkenburg and Peter, 2007). One of these studies argues that the use of the Internet has a positive impact on psychological well-being (McKenna and Bargh, 2000; Shaw and Gant, 2002; Bargh and McKenna, 2004). Some research has proved, for instance, that the Internet could help people with low psychological well-being due to its socialization potential (Bargh and McKenna, 2004). Life satisfaction in relation with the use of Facebook in general has been frequently studied (Blachnio et al., 2016; Kross et al., 2013). Those individuals who are active on Facebook feel more satisfied with their lives as compared to those who do not use Facebook (Valenzuela et al., 2009; Oh et al., 2014). However, scientific evidence is still not conclusive about whether using Facebook enriches users’ lives and makes them feel more satisfied with their lives (Kim and Lee, 2011).

### Use of Facebook and Psychosocial Well-Being

Psychosocial well-being based on the use of social networking sites can be observed according to different aspects that relate to social support, perception of support, affection, company, and sense of community (Oh et al., 2014).

Research on social networking sites has identified social support as one of the most important reasons why people use these sites (Park et al., 2009). The perception of support received from the contacts individuals have on these sites is also a significant predictor for well-being (Vieno et al., 2007). Affection occurs as a result of interpersonal communication (Diener et al., 1991) and it is a combination of moods and emotional states that are considered on social networking sites as “the online assessment of life events” (Diener et al., 1999). Affection received from using social networking sites can be a key predictor for well-being.

Company is another factor that boosts well-being and it is obtained from using social networking sites (Hampton et al., 2011). Being together in these online environments can lead to a sense of community, which is defined as the feeling of belonging to a group or community whose members are perceived as interdependent and similar in terms of characteristics (Sarason, 1974). There are several studies on the relation between the sense of community and the use of social networking sites. Results from these studies vary. However, in general terms, it has been found that when social networking sites are used, the sense of community is a predictor for satisfaction and well-being (Manago et al., 2012).

## Purpose of the Study

Based on the theoretical framework analyzed, we focused our study on Facebook because it is a dominant social networking site. We presumed that DSF can be key variables for Social Work graduates to reach higher PeW. PeW was observed based on the variables of social capital, self-esteem, social satisfaction and SW. The aim was to find out whether Social Work graduates have the necessary digital skills to connect with their equals in a strategic manner, thus allowing them to obtain enough social capital, self-esteem, social satisfaction, and SW.

This assumption led us to verify the following hypothesis:

Social Work graduates who have enough digital skills can achieve social capital (Putnam, 2000), self-esteem (Rosenberg, 1989), life satisfaction (Pavot and Diener, 1993; Diener et al., 1997), and SW (Oh et al., 2014). These four measures are considered to provide well-being.

## Materials and Methods

### Participants

The final strategic sample comprised 126 Social Work graduates from the University of Málaga (Spain). Participants were selected according to their group of age, from 21 to 23, with an average age of 21.6, and due to being considered as digital natives (Prensky, 2001). The assumption is that a series of innate abilities and practices related to the use of technology are conditioned by age, which is why the use of digital means occurs with greater spontaneity. We decided to confirm this hypothesis with Social Work graduates because socialization is considered a core element in Social Work. There were more women in the Social Work program. Analysing the sample according to participants’ characteristics was not the aim of the study. However, we thought the presence of more women could strengthen the analysis given the fact that there is scientific evidence proving that women use Facebook toward achieving a goal and social capital more than men do (Garcia et al., 2016).

### Instruments

In order to analyse the object a survey technique was used. Participants were previously requested their informed consent, thus allowing them to not dill in the questionnaire or leave it incomplete at any given moment.

A questionnaire was drawn up according to two main elements: DSF and PeW. PeW comprises the following variables: BSC and BOSC, self-esteem (SE), life satisfaction (LS), and SW.

To assess the use of Facebook a scale comprising 40 items was drawn up. Items were adapted from the questionnaires of Hargittai and Hsieh (2010), Lampe et al. (2012), Jenkins-Guarnieri et al. (2013), and Ellison et al. (2014). These questionnaires are answered through a five-step Likert-type scale. The dimensions to which questions relate are the following: DSF, strategic DSF, information search on Facebook, use and presence of Facebook in peoples’ lives, actions toward maintaining relations on Facebook and tolerance to diversity on Facebook. Table 1 shows descriptive statistics and Cronbach’s alpha for these dimensions. All of them have good internal consistency, except for tolerance to diversity.

**TABLE 1:** Matrix of correlations, descriptive statistics, and Cronbach’s alpha (α) of digital skill on Facebook and personal well-being.

	1	2	3	4	5	6	7	8	9	10	11	*M*	*SD*	α
Digital skills on Facebook	1	0.418**	0.361**	0.348**	0.466**	0.403**	0.350**	0.271**	0.121	0.090	0.089	27.68	2.81	0.839
Strategic digital skills on Facebook	0.418**	1	0.502**	0.442**	0.508**	0.399**	0.421**	0.311**	0.153	0.129	0.160	37.47	9.42	0.891
Information search on Facebook	0.361**	0.502**	1	0.574**	0.591**	0.446**	0.355**	0.294**	–0.053	–0.009	0.052	11.08	4.37	0.833
Use and presence of Facebook in life	0.348**	0.442**	0.574**	1	0.590**	0.408**	0.363**	0.266**	0.002	0.031	0.032	28.06	9.51	0.914
Actions toward maintaining relations on Facebook	0.466**	0.508**	0.591**	0.590**	1	0.489**	0.429**	0.275**	–0.026	–0.014	0.009	17.68	4.91	0.884
Tolerance to diversity on Facebook	0.403**	0.399**	0.446**	0.408**	0.489**	1	0.328**	0.283**	–0.102	–0.060	–0.090	13.71	2.70	0.439
Bridging social capital	0.350**	0.421**	0.355**	0.363**	0.429**	0.328**	1	0.636**	–0.009	0.007	0.024	35.80	5.90	0.887
Bonding social capital	0.271**	0.311**	0.294**	0.266**	0.275**	0.283**	0.636**	1	0.058	–0.009	0.098	17.61	3.77	0.664
Self-esteem	0.121	0.153	–0.053	0.002	–0.026	–0.102	–0.009	0.058	1	0.474**	0.553**	30.48	3.78	0.786
Satisfaction with life	0.090	0.129	–0.009	0.031	–0.014	–0.060	0.007	–0.009	0.474**	1	0.844**	20.51	2.96	0.804
Psychosocial well-being	0.089	0.160	0.052	0.032	0.009	–0.090	0.024	0.098	0.553**	0.844**	1	31.52	4.85	0.771

**p < 0.01, *p < 0.05.

Social capital is assessed through a five-step scale comprising 14 items adapted from Ellison et al. (2007). They are related to two dimensions: BSC and BOSC. Table 1 shows their statistics. Internal consistency of BSC is high, while it is more moderate for BOSC. Rosenberg’s (1989) self-esteem scale was adapted, which comprises seven items with Likert-type format of five steps and which shows a good internal consistency, as it can be seen in Table 1.

The life satisfaction scale by Diener et al. (1997) and Pavot and Diener (1993) was also used. This scale comprises five Likert-type items of five steps and it shows a good internal consistency, as shown in Table 1.

Finally, the SW scale containing several questions was used (Oh et al., 2014). This scale comprises 13 items, whose statistics are shown in Table 1. It has also a good internal consistency rate.

### Analysis Plan

Analyses were carried out through IBM SPSS Statistics 22. Descriptive statistics and correlations between variables were firstly calculated. Table 1 shows descriptive statistics, Cronbach’s alpha (α) and the matrix of correlations of DSF and PeW, which were used to carry out multiple regression analyses. Five multiple regressions were performed on the five digital skills considered as independent variables and subsequently as dependent variables, BSC and BOSC, self-esteem, life satisfaction, and SW.

## Results

Results obtained are shown in this section. Firstly, Table 1 shows descriptive statistics and correlations. In this table it can be observed that intercorrelations between variables are high and statistically significant, except for self-esteem, satisfaction with life, and SW. Based on this matrix of correlations, the results from multiple regressions carried out are presented.

Table 2 comprises the six variables that refer to Facebook as predictors and BSC and BOSC as dependent variables. Both regressions are significant, although only strategic skills on Facebook predict significantly BSC. However, the effect on BOSC is lower (non-significant). DSF relate to a moderate increase (statistically non-significant) of both types of social capital. There is also a small relation between actions toward maintaining relations and BSC. Tolerance to diversity is weakly related to BSC.

**TABLE 2:** Multiple regression. Digital skills as predictor for bridging and bonding social capital.

	Bridging social capital			Bonding social capital		
		
Use of Facebook	*B*	SE	β	*B*	SE	β
(Constant)	16.563	4.721		7.635	3.238	
Digital skills on Facebook	0.247	0.196	0.118	0.145	0.134	0.108
Strategic digital skills on Facebook	0.129	0.062	0.206**	0.057	0.042	0.144
Information search on Facebook	0.041	0.146	0.030	0.081	0.100	0.095
Use and presence of Facebook in life	0.054	0.065	0.087	0.025	0.045	0.063
Actions toward maintaining relations on Facebook	0.201	0.137	0.167	0.002	0.094	0.003
Tolerance to diversity on Facebook	0.149	0.209	0.068	0.158	0.143	0.113
	*R*^2^ = 0.26			*R*^2^ = 0.109		
	*F* = 7.13, *p* < 0.001			*F* = 3.54, *p* < 0.01		

*p < 0.10, **p < 0.05, ***p < 0.01.

Regressions were not statistically significant (see Table 3). Locally, it must be noted that strategic DSF increase self-esteem, life satisfaction and SW. Tolerance to diversity decreases self-esteem, SW (both significantly), and life satisfaction (moderately). The remaining relations are non-significant.

**TABLE 3:** Multiple regression. Digital skills as predictor for self-esteem, satisfaction with life, and psychosocial well-being.

	Self-esteem			Satisfaction with life			Psychosocial well-being		
			
Use of Facebook	*B*	SE	β	*B*	SE	β	*B*	SE	β
(Constant)	26.125	3.374		18.008	2.702		28.308	4.382	
Digital skills on Facebook	0.222	0.140	0.165	0.116	0.112	0.110	0.170	0.182	0.099
Strategic digital skills on Facebook	0.100	0.044	0.249**	0.059	0.035	0.189*	0.110	0.057	0.214**
Information search on Facebook	–0.106	0.104	–0.122	–0.041	0.083	–0.060	0.042	0.135	0.038
Use and presence of Facebook in life	0.012	0.047	0.030	0.016	0.037	0.050	0.003	0.060	0.006
Actions toward maintaining relations on Facebook	–0.065	0.098	–0.084	–0.055	0.078	–0.092	–0.074	0.127	–0.075
Tolerance to diversity on Facebook	–0.259	0.149	−0.184*	–0.141	0.119	–0.128	–0.355	0.194	−0.197*
	*R*^2^ = 0.086			*R*^2^ = 0.045			*R*^2^ = 0.62		
	*F* = 1.86, *p* = 0.09			*F* = 0.938, *p* = 0.47			*F* = 1.31, *p* = 0.25		

*p < 0.10, **p < 0.05, ***p < 0.01.

## Discussion

In little more than a decade, social networking sites have shaken up the way in which we interact and connect to each other. Their effect on PeW has been analyzed, leading to diverse results. Our approach suggested the novelty of finding out whether the different ways of participating in these socialization platforms can lead to achieving PeW. In order to do so, we considered digital skills as a relevant factor. Results show that when young people, in this case Social Work graduates, have the necessary DSF, they tend to establish and maintain strategic relations that provide key information and improve their social positions. This means that when young people know how to identify appropriate information, when they connect to their closest circle but also to those who they see occasionally, when they plan activities with opinions under their own initiative, when they participate in groups deliberately to achieve a specific goal, when they know who to add as a friend and who not to have amongst their contacts, based on the information shared by these, and when they know how to work together through Facebook, they reach social capital. Having strategic DSF also improves young graduates’ self-esteem, life satisfaction and SW. This means that when young people acquire necessary strategic skills to use Facebook to achieve a specific goal, they feel better as they have a more positive image of themselves. The sense of security provided by strategic digital skills can also encourage them to feel more satisfied with their lives. The psychosocial variable, which considers indicators of social support, perception of support, affection, company and sense of community has also been observed to increase when young people have such digital skills.

However, establishing relations and reaching social capital, which allows reaching more diverse information, does not imply being more tolerant to diversity. This suggests that higher tolerance to diversity makes individuals be exposed with higher intensity in the network to discrepant content that is contrary to their own opinions. This can lead to unease due to seeing their beliefs questioned and feeling that those other individuals are right and they are not. In sum, it seems that strategic digital skills can influence the well-being of Social Work young graduates.

## Limitations

This study focuses on a group of very specific subjects, that is, Social Work young graduates. Despite the fact that such sample was deliberately chosen, we consider it convenient to broaden it and include young people from other disciplines, so a more diverse sample with varied sociodemographic characteristics can be studied. In future studies, we must broaden the sample in order to find out if scientific evidence obtained from the present study apply to larger samples.

## Conclusion

Social networking sites have become socialization tools that allow reaching information and establishing networks with certain orientation toward achieving specific goals. Promoting strategic digital skills from the educational Social Work is essential, as it allows students to understand how to use these tools for their own benefit and for the process of digital inclusion that they will have to carry out. Amongst young adults, relations with their peers on social networking sites are important for obtaining benefits in the offline reality, such as social capital and personal and SW (Steinfield et al., 2008). Taking into consideration the academic context, Brown and Adler (2008) note that adopting these means requires a radical swift in the pedagogical approach with “revolutionary” consequences for academic institutions or, at least, to be considered by teachers. Junco (2014) noted that using these social means in higher education can lead to reconnect academic institutions with new generations of students. Increasing the use of social networking sites in education would make students be more engaged and determined with their studies (Junco, 2012). This is the reason why more and more researchers and education staff are using social networking sites for the academic processes of teaching-learning (Bosch, 2009). This is even more important for disciplines such as Social Work and other social sciences, in which socialization and community promotion are core elements. It is essential to incorporate these skills in academic curricula in order to boost the benefits and mitigate the harm of using social networking sites.

## Data Availability Statement

The datasets generated for this study are available on request to the corresponding author.

## Ethics Statement

The studies involving human participants were reviewed and approved by the Ethics Committee of University of Málaga. The patients/participants provided their written informed consent to participate in this study.

## Author Contributions

All authors listed have made a substantial, direct and intellectual contribution to the work, and approved it for publication.

## Conflict of Interest

The authors declare that the research was conducted in the absence of any commercial or financial relationships that could be construed as a potential conflict of interest.
